# Amyloid β Regulates the Expression and Function of AIP1

**DOI:** 10.1007/s12031-014-0310-y

**Published:** 2014-07-02

**Authors:** Huaiming Wang, Lijing Fan, Hong Wang, Xixin Ma, Zhongde Du

**Affiliations:** 1 Department of Neurology, The 89th Hospital of People’s Liberation Army, 256 Beigong west Street, Weifang, 261045 Shandong Province China; 2 Department of medicine, Shandong Province Weifang Brain hospital, Weifang, China

**Keywords:** Alzheimer’s disease, Aβ1-42, Apoptosis signal-regulating kinase 1–interacting (ASK1-interacting) protein-1 (AIP1), Apoptosis signal-regulating kinase 1 (ASK1), Apoptosis

## Abstract

Apoptosis signal-regulating kinase 1–interacting (ASK1-interacting) protein-1 (AIP1) is a newly identified novel member of the Ras GTPase-activating protein family, which has been implicated in cell growth inhibition and cell apoptosis. However, the effects of AIP1 in Alzheimer’s disease (AD) are unknown. In the present study, we found that AIP1 was elevated in the brain of AD Tg2576 mice and Aβ1-42 treated brain cerebral microvascular endothelial cells (CECs). Aβ1-42 treatment induced the interaction of AIP1 and apoptosis signal-regulating kinase 1 (ASK1), which led to dissociation of ASK1 and its inhibitor 14-3-3. Dissociation of ASK1 from 14-3-3 leads to ASK1 activation. Indeed, Aβ1-42 dephosphorylated ASK1 at Ser-967, suggesting that Aβ1-42 increased ASK1 activity. Importantly, disassociation of ASK1 and 14-3-3 induced by Aβ1–42 could be rescued by silence of AIP1. In addition, down-regulation of AIP1 also led to attenuation of the activation of JNK, as well as p53, downstream signaling targets of ASK1. AIP1 silencing attenuated the pro-apoptotic effects of Aβ1-42 on CECs. We propose that AIP1 mediates Aβ induced ASK1 activation by facilitating dissociation of 14-3-3, suggesting a novel mechanism for Aβ-induced apoptosis in CECs.

## Introduction

Alzheimer's disease (AD) is neuropathologically characterized by intracellular neurofibrillary tangles (NFTs) and extracellular senile plaques (Roberson and Mucke 2006). The main component of extracellular senile plaques is β-amyloid (Aβ) (Buoso et al. 2010). Aβ has been reported as a primary mediator of neuronal degeneration in AD (Yankner et al. 1989; Sheng et al. 2009a). Multiple lines of evidence suggest that Aβ induces apoptosis not only in neurons, but also in cerebral endothelial cells (CECs) (Yin et al. 2002). CECs play a critical role in blood–brain barrier (BBB) to shield the brain from damage by harmful circulating toxins or deleterious cellular elements. Currently, cerebrovascular pathology and AD are strongly linked. Aβ deposition in cerebrovascular surface forms cerebral amyloid angiopathy (CAA), which has been proved to play a critical role in the progress of AD (Yamada and Naiki 2012). As a result, CEC apoptosis appears to be a contributing factor in Aβ-induced cerebrovascular degeneration. However, the mechanisms underlying the CEC apoptosis are still needed to be elucidated.

Apoptosis signal-regulating kinase 1–interacting (ASK1-interacting) protein-1 (AIP1) is a recently identified novel member of the Ras GTPase-activating protein family. It is also known as DAB2-interacting protein (DAB2IP) and has been implicated in cell growth inhibition and cell apoptosis (Chen et al. 2005). AIP1 has been reported to function as a positive regulator in cell apoptosis by mediating activation of the apoptotic kinase apoptosis signal-regulating kinase 1 (ASK1). AIP1 binds preferentially to dephosphorylate ASK1 and recruited AIP1 that enhances ASK1-induced JNK activation (Zhang et al. 2003a). Previous studies have demonstrated that AIP1 expression is often down-regulated in various human cancers, suggesting that AIP1 serves as an inhibitor of cell survival and growth (Yano et al. 2004). However, whether AIP1 is involved in the neurodegenerative process of AD is still unknown. In this study, we aimed to determine whether alternations in AIP1 signaling contribute to Aβ-induced CEC death.

## Materials and Methods

### Animal Models

Tg2576 mice (18–24 months) overexpressing human APP695 with the “Swedish” mutation develop memory deficits and plaques with age were used in this study. The transgenic mice were purchased from Taconic Farms (Germantown, USA). Mice were maintained on mixed C57Bl6/SJL background by mating heterozygous Tg2576 males to C57Bl6/SJL F1 females. Age-matched non-transgenic littermates of the Tg2576 mice (Control) were used as control animals. All experimental procedures were carried out under protocol approved by the Institutional Animal Care and Use Committee in the 89th Hospital of People's Liberation Army and were in accordance with the 89th Hospital of People's Liberation Army guidelines for the care and use of laboratory animals.

### Cell Culture, Treatment, and Transfection

The human brain cerebral microvascular endothelial cells (CECs) were purchased from Cell Systems and cultured in EBM-2 media with supplemental growth factors according to manufacturer’s instructions (Shi et al. 2013). Aβ1-42 (American Peptide, USA) was dissolved in Hexafluoroisopropanol (Sigma, USA) for 2 days at room temperature (RT), and the lyophilized peptide was dissolved in dimethylsulfoxide (DMSO). Ten to twenty micromolar of Aβ1-42 was used to treat cells for 48 h. AIP1 knockdown experiments were conducted by transfecting with nonspecific or AIP1-specific small interfering RNA (siRNA) (50 nM, Santa Cruz) using Lipofectamine RNAiMAX (Invitrogen).

### Real Time Quantitative Polymerase Chain Reaction (PCR)

Total RNA was extracted from mouse whole brain tissue and CECs with TRIzol Reagent (Invitrogen). Two microgram target RNA in each group was converted into complementary DNA (cDNA) by using iScript™ RT-PCR Kit (Bio-rad, USA). The synthesized cDNA products were used for real time PCR with SYBR Green Supermix (Bio-Rad Laboratories) as the fluorescent DNA intercalating agent. The following primers were used in this study: AIP1, 5′-CCTGCGCGTATCAGTCCTTACCA-3′ (forward); 5′-GGGTTCAGAGCCCTCCTC-3′ (reverse); GAPDH, 5′-GGAGAAGGCTGGGGCTCAT-3′ (forward); 5′-TGATGGCATGGACTGTGGTC-3′ (reverse).

### Western Blot Analysis

For immunoprecipitation assays, cells were lysed by cell lysis buffer containing 50 mM Tris–HCl (pH 7.5), 150 mM NaCl, 0.1 % Nonidet P-40, 1 mM dithiothreitol, protease inhibitor mixture, sodium orthovanadate, and sodium fluoride. Twenty millimolar sodium molybdate was used to stabilize Hsp90 protein interactions. A total of 5 μg of anti-HIF-1α antibody (Novus Biological, USA) was incubated with 1 mg whole cell lysates to form the antigen-antibody complex overnight at 4 °C. The formed antigen-antibody complex was incubated with protein A/G-agarose beads (Santa Cruz) for 2 h at 4 °C. After washed with phosphate-buffered saline four times, the beads were eluted in SDS sample buffer. Eluted protein samples were subjected to 10 % sodium dodecyl sulfate polyacrylamide gel electrophoresis (SDS-PAGE) and electrotransferred onto polyvinylidene fluoride (PVDF) membranes (Millipore, USA). PVDF membranes were blocked in blocking solution (5 % nonfat dry milk in tris-buffered saline containing 0.1 % Tween 20) for 2 h at room temperature. Then membranes were sequentially incubated overnight with primary antibody and horse radish peroxidase (HRP)-conjugated secondary antibody for another 2 h. Blots were developed with chemiluminescence technique (Santa Cruz Biotechnology, USA) according to the manufacturer’s instructions.

### TUNEL Assay and DAPI Staining

DNA fragmentation is a characteristic hallmark of apoptosis. The patterns of apoptosis were indexed by the Terminal deoxynucleotidyl transferase dUTP nick end labeling (TUNEL). Briefly, CECs were plated in Lab-Tek® Chamber Slides (Nalgene Nunc, USA) and incubated in the indicated medium. Upon completion of the indicated treatment, apoptosis was determined by a commercially available kit (Promega, Madison, WI, USA) according to the manufacturer’s instructions. Nuclei were counterstained with nuclear-specific dye 4, 6-diamidino-2-phenylindole (DAPI). Fluorescence signals were recorded by using a fluorescence microscope and a digital camera.

### Statistical Analysis

All quantitative data are presented as mean ± SEM from at least three separate experiments. One-way analysis of variance (ANOVA) was used to assess statistical significance of differences among treatment groups. *P* < 0.05 was considered statistically significant.

## Results

**Fig. 1:**
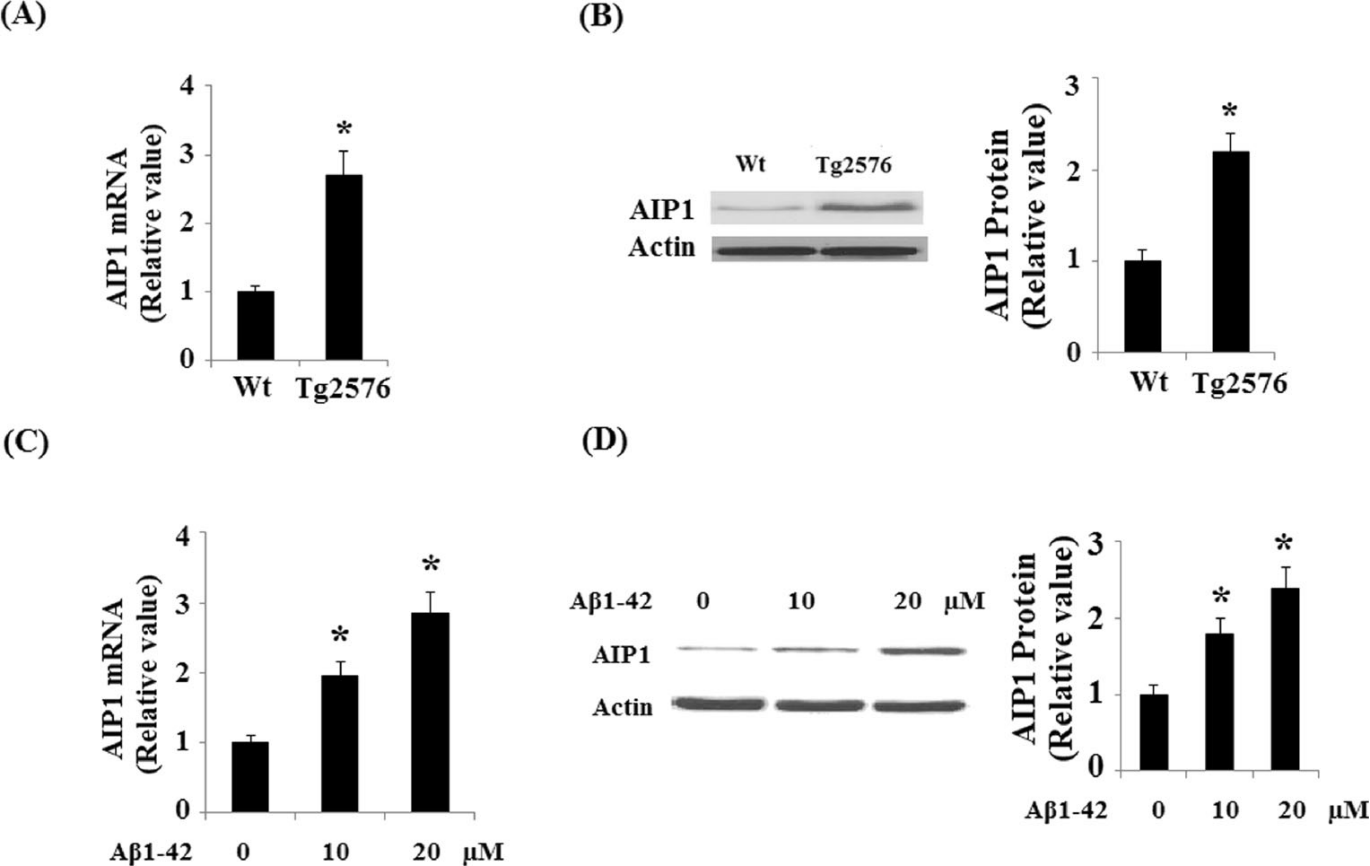
The expression level of A1P1 was increased in the brain of Tg2576 mice and Aβ1-42 treated primary cerebral endothelial cells (CECs). **a** Real time PCR analysis revealed that the mRNA level of A1P1 was significantly increased in Tg2576 brains compared with controls (*p < 0.01 vs. controls, Student’s *t* test, *n* = 6); **b** representative immunoblot and quantification analysis revealed that the protein level of A1P1 was significantly increased in Tg2576 brains compared with age-matched controls (*p < 0.01 vs. control, Student’s *t* test, *n* = 6); actin was used as an internal loading control. **c** CECs were treated with Aβ1-42 (10 or 20 μM) for 48 h, real time PCR analysis revealed that the mRNA level of AIP1 was significantly increased after Aβ1-42 treatment (*p < 0.01 vs. untreated control, ANOVA, *n* = 4); **d** CECs were treated with Aβ1-42 (10 or 20 μM) for 48 h, representative immunoblot and quantification analysis revealed that the protein level of AIP1 was significantly increased after Aβ1-42 treatment (*p < 0.01 vs. untreated control, ANOVA, *n* = 4)

In order to examine whether the expression of AIP1 was changed in AD, we investigated the expression levels of AIP1 in the brains of Tg2576 mice at messenger RNA (mRNA) levels and protein levels. As shown in Fig. 1a, real time PCR studies showed a significant up-regulation in AIP1 mRNA level in Tg2576 mice compared to age-matched controls. Western blot analysis also demonstrated that the protein level of AIP1 was significantly elevated in the brains of Tg2576 mice (Fig. 1b).

It was reported that AIP1 is highly expressed in the vascular endothelium (Zhang et al. 2008a). As Aβ was extensively associated with vascular endothelium dysfunction in AD, we investigated the effect of Aβ1-42 in the expression of AIP1 in human primary cerebral endothelial cells (CECs). Isolated CECS was treated with Aβ1-42 for 48 h, and the Western blot result indicated that Aβ1-42 treatment led to a significant elevation in AIP1 expression at both mRNA levels (Fig. 1c) and protein levels (Fig. 1d).

**Fig. 2:**
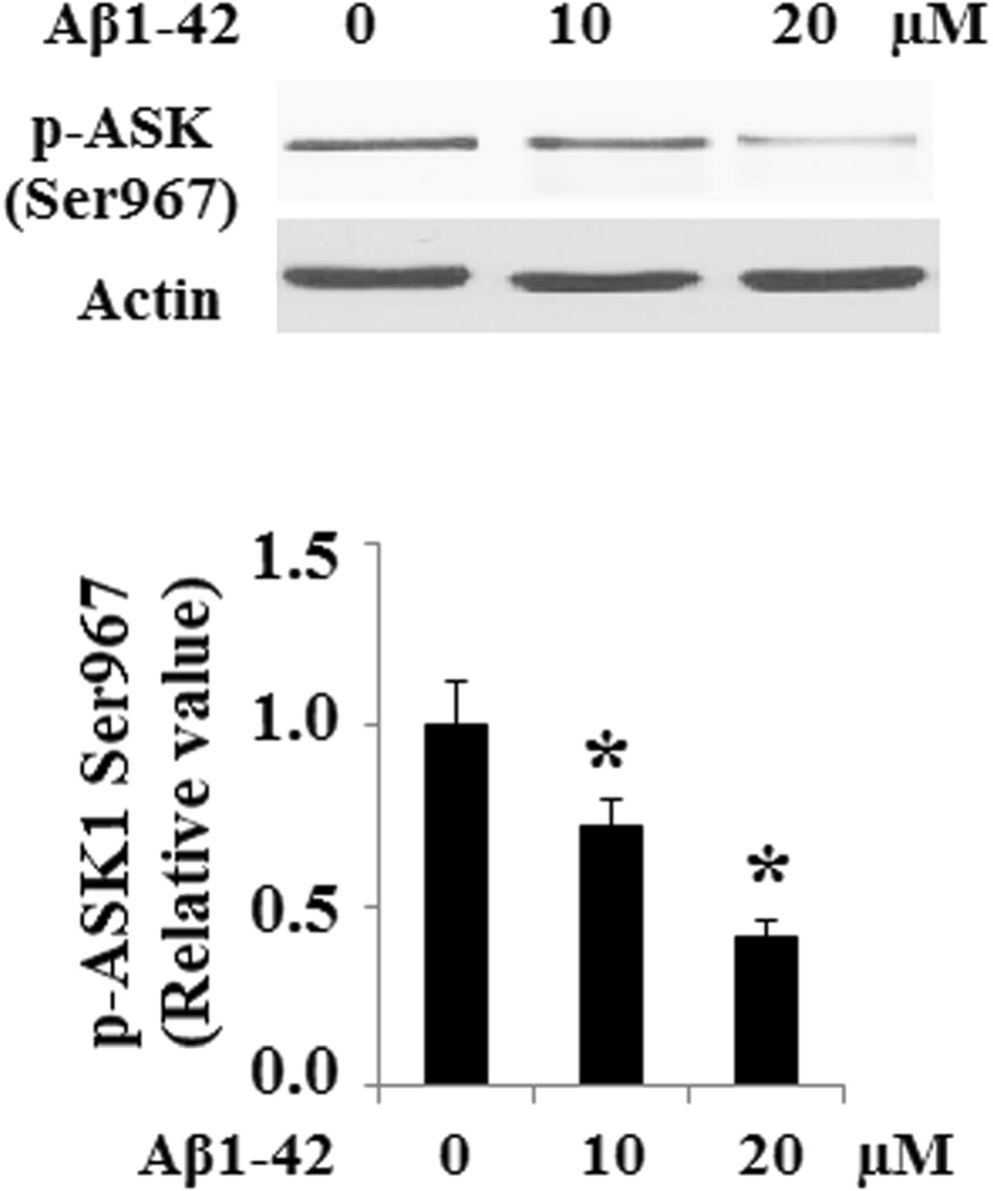
Aβ1-42-induced phosphorylation of ASK at Ser967. Phosphorylated levels of ASK at Ser967 were examined by Western blot analysis in CECs in presence of Aβ1-42 (*p < 0.01 vs. untreated control, ANOVA, *n* = 3–4)

**Fig. 3:**
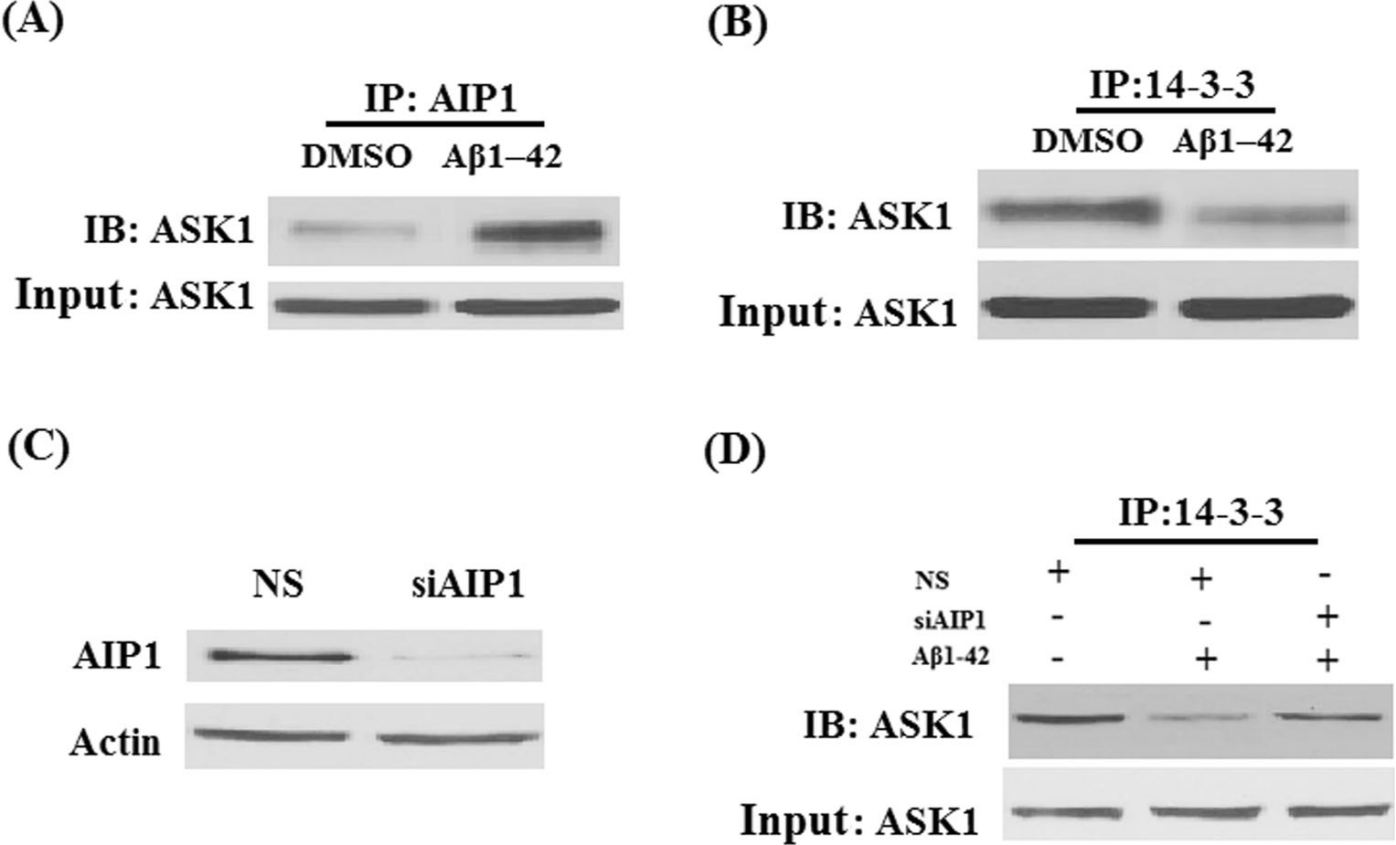
Aβ1-42 promotes the interaction between AIP1 and ASK1, but inhibits the interaction between 14-3-3 and ASK1. **a** After treated with Aβ1-42 for 48 h, the cell lysates were immunoprecipitated (IP) by anti-AIP1 antibody. The precipitate was immunoblotted (IB) with anti-ASK1; representative blots of four independent experiments are shown. **b** After treated with Aβ1-42 for 48 h, the cell lysates were immunoprecipitated (IP) by anti-14-3-3 antibody. The precipitate was immunoblotted (IB) with anti-ASK1. Representative blots of four independent experiments are shown. **c** The expression of AIP1 was knocked down using small RNA interferences, and Western blot analysis confirmed the successful knockdown of AIP1; **d** Immunoprecipitated (IP) experiment demonstrates that knockdown of AIP1 attenuated the disassociation between 14-3-3 and ASK1. Representative blots of four independent experiments are shown

Aβ1-42 dephosphorylated ASK1 at Ser-967, suggesting that Aβ1-42 increased ASK1 activity (Fig. 2). Interaction of AIP1 with endogenous ASK1 was determined by co-immunoprecipitation with primary anti-AIP1 antibody followed by Western blot with ASK1 antibody. And the result indicated that Aβ1-42 treatment induced the interaction of AIP1 and ASK1 (Fig. 3a). 14-3-3 is an inhibitor of ASK1. When binds to 14-3-3, ASK1 stays inactive. It was previously shown that 14-3-3 associated with ASK1 via pSer-967 (Subramanian et al. 2004). Dissociation of ASK1 from 14-3-3 leads to ASK1 activation, initialing intracellular apoptosis pathways. Interestingly, co-immunoprecipitation results revealed that Aβ1-42 abolished the association of 14-3-3 and ASK1 (Fig. 3b). In order to examine whether AIP1 mediated Aβ1-42-induced disassociation of ASK1 and 14-3-3, the association of 14-3-3 and ASK1 was investigated following siRNA mediated down-regulation of AIP1. The successful silence of AIP1 was shown in Fig. 3c. Importantly, disassociation of ASK1 and 14-3-3 induced by Aβ1-42 could be rescued by silence of AIP1 (Fig. 3d).

**Fig. 4:**
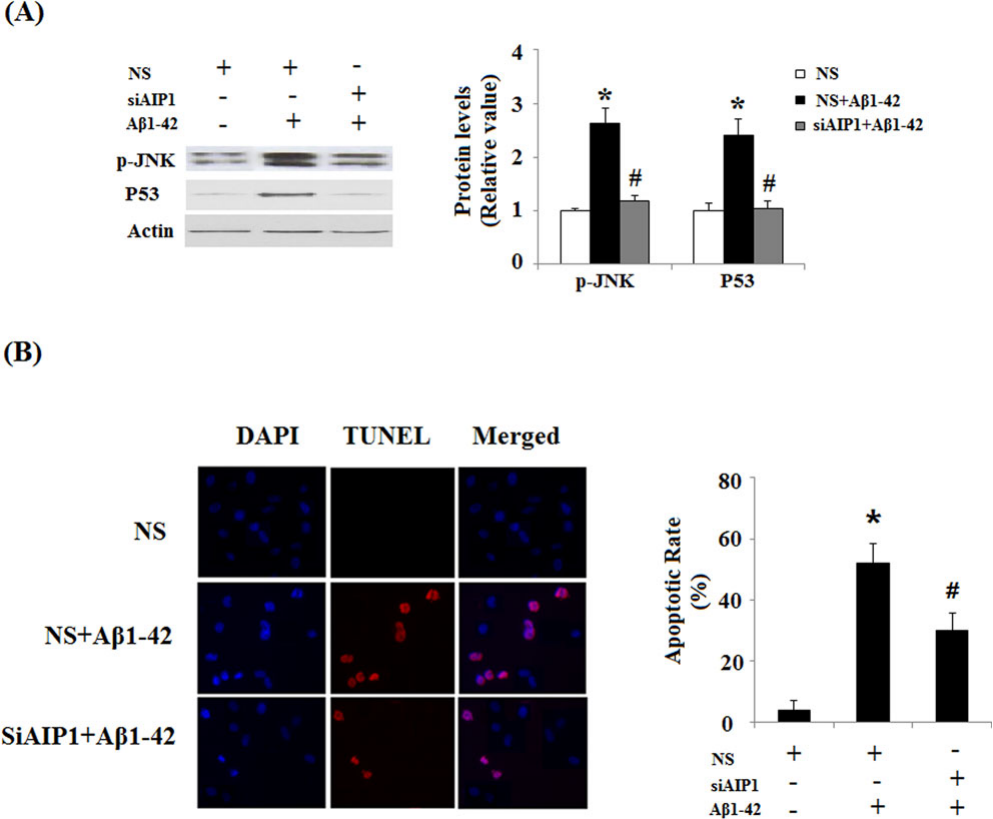
Knockdown of AIP1 by small RNA interference mitigated Aβ1-42 induced activation of JNK, induction of P53, and apoptosis. **a** After transfected with AIP1 siRNA, cells were incubated with Aβ1-42 (20 μM) for 48 h, immunoblot and quantification analysis revealed that Aβ1-42-induced up-regulation of phosphorylated JNK and P53 could be attenuated by knockdown of AIP1 (*p < 0.01 vs. Non-treatment; #*p* < 0.01 vs. Aβ1–42 treatment, *n* = 4–5). **b** Silence of AIP1 prevents Aβ1-42 (20 μM)-induced apoptosis in CECs. Cells were stained using a TUNEL Assay Kit. Nuclear DNA was stained with DAPI (ANOVA; *p < 0.001 vs. non-treated control; #*p* < 0.001 vs. Aβ1-42 treated group, *n* = 4–5)

Activated ASK1 plays a critical role in apoptosis by stimulating the downstream signaling events, especially JNK activation. Indeed, down-regulation of AIP1 also led to attenuation of the activation of JNK, as well as the expression of p53 (Fig. 4a), downstream signaling targets of ASK1, suggesting that inhibition of AIP1 could prevent the activated apoptotic signaling cascade induced by Aβ1-42. In order to determine whether elevated AIP1 contributed to pro-apoptotic effects of Aβ in CECs cells, TUNEL staining was used to detect the apoptosis patterns after Aβ treatment. Five micromolar of Aβ1-42 significantly promoted apoptosis in mouse primary CECs. However, AIP1 silencing attenuated the pro-apoptotic effects of Aβ1-42 (Fig. 4b).

## Discussion

AIP1 was initially identified as a binding partner for apoptosis signaling kinase-1 (ASK1). It was reported AIP1 functions as an adaptor molecule in TNF signaling (Zhang et al. 2003b). A recent study demonstrated that AIP1 activated ASK1 by recruiting phosphatase PP2A to dephosphorylate the 14-3-3 binding site on ASK1, leading to dissociation of this inhibitory protein from the enzyme, thereby allowing activation by the TNFR1 signaling complex (Min et al. 2008). As highly expressed in endothelial cells, the biological function of AIP1 in vascular EC has been widely studied. AIP1 was reported to function as an inhibitor of VEGFR2 signaling in vascular EC surface, regulating angiogenesis and lymphangiogenesis (Zhang et al. 2008b). However, to the best of our knowledge, there is no report on the function of AIP1 in AD. So far, Aβ has been implicated as the primary neurotoxic mediator in the pathogenesis of AD (Sheng et al. 2009b). The mechanisms underlying Aβ-induced apoptosis are complex. It was considered as a multifactorial process, including excessive formation of reactive oxygen species, alteration of intracellular calcium homeostasis (Demuro et al. 2010), and mitochondrial dysfunction (Leuner et al. 2012) and caspases activation (Mattson 2006). However, the precise molecular mechanism responsible for the apoptotic action of Aβ remains to be fully characterized. Here, we reported the new mechanisms underlying Aβ-induced CECs apoptosis by investigating the roles of AIP1. The level of AIP1 was increased in the brains of AD Tg2576 mice, suggesting a potential role of AIP1 in the pathogenesis of AD. Indeed, Aβ1-42 treatment promoted the expression of AIP1. The elevated AIP1 binds to the dephosphorylated ASK1, activating as ASK1 signaling pathway by disassociating ASK1 from its inhibitor 14-3-3. ASK1 is the activator of JNK (Matsuzawa and Ichijo 2008). Activated ASK1 has been associated with neurotoxicity of Aβ in CECs (Hsu et al. 2007). Moreover, activated JNK has been extensively involved in the apoptotic effect of Aβ (Borsello and Forloni 2007). Inhibition of AIP1 attenuated the activation of ASK1/JNK/p53 induced by Aβ1-42. More importantly, AIP1 silencing using siRNA rescued Aβ1-42-induced apoptosis. These data suggest that up-regulation of AIP1 might be a novel regulator, involved in the neurotoxicity of Aβ1-42.

ASK1 is a member of the mitogen-activated protein kinase kinase kinase (MAPKKK) family which is the upstream signaling molecular of the JNK and p38 MAPK signaling cascades (Saitoh et al. 1998). Studies have reported that ASK1 is related to various cellular responses including oxidative stress, cell survival, and apoptosis (Takeda et al. 2000). Importantly, Aβ neurotoxicity is related to the activation of ASK1 (Song et al. 2014). Aβ can activate ASK1, leading to the generation of ROS and ER stress-induced JNK activation (Kadowaki et al. 2005). Consistent with our current study, a recent study reported that the activation of the ASK1-MKK3/6-p38MAPK signaling cascade triggered Aβ-induced cell death in cerebral endothelial cells (Hsu et al. 2007). Also, Aβ-induced apoptosis in SH-SY5Y cells is mediated by activation of the ASK1 cascade (Akterin et al. 2006). In addition, the dimerization of the cytoplasmic domains of APP induces ASK1- and JNK-dependent apoptosis in neuronal cells (Hashimoto et al. 2003; Maruoka et al. 2003). Abnormal tau phosphorylation is another essential pathological event that occurs in AD. Peel and colleagues demonstrated that the activation of ASK1 is involved in tau phosphorylation (Peel et al. 2004). Multiple lines of evidence have shown that ASK1 is involved in insulin signal, which plays an important role in AD pathology such as cognitive impairment (Aguirre et al. 2000; Craft et al. 1999). Taken together, ASK1 is associated with various mechanisms in AD pathology. It is suggested to be a potential therapeutic target for AD.

Based on these observations, we concluded that AIP1 mediates Aβ induced ASK1 activation by facilitating dissociation of 14-3-3, suggesting a novel mechanism for Aβ-induced apoptosis in CECs.

## References

[CR1] Aguirre V, Uchida T, Yenush L (2000). The c-Jun NH(2)-terminal kinase promotes insulin resistance during association with insulin receptor substrate-1 and phosphorylation of Ser(307). J Biol Chem.

[CR2] Akterin S, Cowburn RF, Miranda-Vizuete A (2006). Involvement of glutaredoxin-1 and thioredoxin-1 in beta-amyloid toxicity and Alzheimer’s disease. Cell Death Differ.

[CR3] Borsello T, Forloni G (2007). JNK signalling: a possible target to prevent neurodegeneration. Curr Pharm Des.

[CR4] Buoso E, Lanni C, Schettini G (2010). beta-Amyloid precursor protein metabolism: Focus on the functions and degradation of its intracellular domain. Pharmacol Res.

[CR5] Chen H, Tu SW, Hsieh JT (2005). Downregulation of human DAB2IP gene expression mediated by polycomb Ezh2 complex and histone deacetylase in prostate cancer. J Biol Chem.

[CR6] Craft S, Asthana S, Newcomer JW (1999). Enhancement of memory in Alzheimer disease with insulin and somatostatin, but not glucose. Arch Gen Psychiatry.

[CR7] Demuro A, Parker I, Stutzmann GE (2010). Calcium signaling and amyloid toxicity in Alzheimer disease. J Biol Chem.

[CR8] Hashimoto Y, Niikura T, Chiba T (2003). The cytoplasmic domain of Alzheimer’s amyloid-beta protein precursor causes sustained apoptosis signal-regulating kinase 1/c-Jun NH2-terminal kinase-mediated neurotoxic signal via dimerization. J Pharmacol Exp Ther.

[CR9] Hsu MJ, Hsu CY, Chen BC (2007). Apoptosis signal-regulating kinase 1 in amyloid beta peptide-induced cerebral endothelial cell apoptosis. J Neurosci.

[CR10] Kadowaki H, Nishitoh H, Urano F (2005). Amyloid beta induces neuronal cell death through ROS-mediated ASK1 activation. Cell Death Differ.

[CR11] Leuner K, Müller WE, Reichert AS (2012). From mitochondrial dysfunction to amyloid beta formation: Novel insights into the pathogenesis of Alzheimer's disease. Mol Neurobiol.

[CR12] Maruoka S, Hashimoto S, Gon Y (2003). ASK1 regulates influenza virus infection-induced apoptotic cell death. Biochem Biophys Res Commun.

[CR13] Matsuzawa A, Ichijo H (2008). Redox control of cell fate by MAP kinase: physiological roles of ASK1-MAP kinase pathway in stress signaling. Biochim Biophys Acta.

[CR14] Mattson MP (2006). Neuronal life-and-death signaling, apoptosis, and neurodegenerative disorders. Antioxid Redox Signal.

[CR15] Min W, Lin Y, Tang S (2008). AIP1 recruits phosphatase PP2A to ASK1 in tumor necrosis factor-induced ASK1-JNK activation. Circ Res.

[CR16] Peel AL, Sorscher N, Kim JY (2004). Tau phosphorylation in Alzheimer’s disease: Potential involvement of an APP-MAP kinase complex. Neuromol Med.

[CR17] Roberson ED, Mucke L (2006). 100 years and counting: Prospects for defeating Alzheimer's disease. Science.

[CR18] Saitoh M, Nishitoh H, Fujii M (1998). Mammalian thioredoxin is a direct inhibitor of apoptosis signal-regulating kinase (ASK) 1. EMBO J.

[CR19] Sheng B, Song B, Zheng Z (2009). Abnormal cleavage of APP impairs its functions in cell adhesion and migration. Neurosci Lett.

[CR20] Sheng B, Gong K, Niu Y (2009). Inhibition of gamma-secretase activity reduces Abeta production, reduces oxidative stress, increases mitochondrial activity and leads to reduced vulnerability to apoptosis: Implications for the treatment of Alzheimer's disease. Free Radic Biol Med.

[CR21] Shi H, Sheng B, Zhang F (2013). Kruppel-like factor 2 protects against ischemic stroke by regulating endothelial blood brain barrier function. Am J Physiol Heart Circ Physiol.

[CR22] Song J, Park KA, Lee WT (2014). Apoptosis signal regulating kinase 1 (ASK1): Potential as a therapeutic target for Alzheimer's disease. Int J Mol Sci.

[CR23] Subramanian RR, Zhang H, Wang H (2004). Interaction of apoptosis signal-regulating kinase 1 with isoforms of 14-3-3 proteins. Exp Cell Res.

[CR24] Takeda K, Hatai T, Hamazaki TS (2000). Apoptosis signal-regulating kinase 1 (ASK1) induces neuronal differentiation and survival of PC12 cells. J Biol Chem.

[CR25] Yamada M, Naiki H (2012). Cerebral amyloid angiopathy. Prog Mol Biol Transl Sci.

[CR26] Yankner BA, Dawes LR, Fisher S (1989). Neurotoxicity of a fragment of the amyloid precursor associated with Alzheimer’s disease. Science.

[CR27] Yano M, Toyooka S, Tsukuda K (2004). Aberrant promoter methylation in human DAB2 interactive protein (hDAB2IP) gene in breast cancer. Clin Cancer Res.

[CR28] Yin KJ, Lee JM, Chen SD (2002). Amyloid-beta induces Smac release via AP-1/Bim activation in cerebral endothelial cells. J Neurosci.

[CR29] Zhang R, He X, Liu W (2003). AIP1 mediates TNF-α induced ASK1 activation by facilitating dissociation of ASK1 from its inhibitor 14-3-3. J Clin Invest.

[CR30] Zhang R, He X, Liu W (2003). AIP1 mediates TNF-alpha-induced ASK1 activation by facilitating dissociation of ASK1 from its inhibitor 14-3-3. J Clin Invest.

[CR31] Zhang H, He Y, Dai S (2008). AIP1 functions as an endogenous inhibitor of VEGFR2-mediated signaling and inflammatory angiogenesis in mice. J Clin Invest.

[CR32] Zhang H, He Y, Dai S (2008). AIP1 functions as an endogenous inhibitor of VEGFR2-mediated signaling and inflammatory angiogenesis in mice. J Clin Invest.

